# A rare case report of anal canal adenocarcinoma

**DOI:** 10.1097/MD.0000000000027083

**Published:** 2021-09-17

**Authors:** Xiaowei Song, Huimin Zhao, Yongping Yang, Linxian Zhao, Yongqing Zhao, Jiannan Li

**Affiliations:** a Department of General Surgery, The Second Hospital of Jilin University, Changchun, Jilin, China; b Operating Theater and Department of Anesthesiology, The Second Hospital of Jilin University, Changchun, Jilin, China.

**Keywords:** anal canal adenocarcinoma, abdominal perineal resection, chemotherapy, computer tomography, magnetic resonance imaging, radiotherapy

## Abstract

**Rationale:**

Anal canal adenocarcinoma is a kind of rare malignant tumor of the intestinal tract with a low incidence rate.

**Patient concerns:**

A 42-year-old man came to our department with anal tenderness accompanied by intermittent drainage of mucus discharge for 2 weeks.

**Diagnoses:**

The computer tomography showed a strip-shaped high-density shadow in the rectal wall. The magnetic resonance imaging showed a cyst-like mass of about 33 × 57 × 30 mm in the anal area. The lesion penetrated the anal canal, and plaque-shaped high signal shadow can be seen in the left side of the anus. The intraoperative pathology indicated the mass as anal canal adenocarcinoma.

**Interventions:**

The abdominal perineal resection was performed for this patient. The postsurgical pathology showed that the tumor was anal canal adenocarcinoma with large amounts of mucus.

**Outcomes:**

The patient recovered well and was discharged from our department at 12th day post-surgery. This patient received further pelvic radiotherapy.

**Lessons:**

Anal canal adenocarcinoma is a kind of malignant tumor that is extremely rare clinically. Computer tomography, magnetic resonance imaging, coloscopy, and histopathology are vital for the diagnosis of anal canal adenocarcinoma. Comprehensive treatment, including abdominal perineal resection, radiotherapy, and chemotherapy, is important for the treatment of anal canal adenocarcinoma.

## 1 Introduction

Anal canal cancer is a kind of rare malignant tumor of the intestinal tract. Squamous cell carcinoma accounts for 75% to 80% of anal canal cancers, and others are mainly adenocarcinoma and adenosquamous carcinoma.^[1]^ With the development of social culture and living behavior, the incidence of anal canal cancer has increased in the past decades and may continue increasing in the future. Anal canal adenocarcinoma mainly originates from the anal canal epithelium and includes the primary adenocarcinomas which derive from the mucosa surface, anal glands, and fistula.^[2,3]^ Anal canal adenocarcinoma is very rare in clinic, and the correct diagnosis mainly depends on the histopathology analysis. In this study, we reported a rare case of anal canal adenocarcinoma that was diagnosed with anal fistula first. But the rapid intraoperative pathology confirmed the diagnosis of anal canal cancer, and radical surgical resection was further performed for this patient.

## 2 Case report

This study was approved by the Ethics Committee and Institutional Review Board of the Second Hospital of Jilin University, Changchun, China. The patient provided informed consent for publication of the case.

A 42-year-old man came to our department with anal tenderness accompanied by intermittent drainage of mucus discharge for 2 weeks. The patient had anal pain with no obvious inducement 2 weeks ago. The patient also complained about increased defecation frequency (7–8 times a day) with a sense of urgency. The fecal volume was small, and a small amount of dark red bloody and a large number of mucus secretions on the surface of the defecation can be found. This patient didn’t receive systematic treatment.

Medical history: The patient underwent radical resection of anal fistula 3 years ago. He denied any history of hypertension, diabetes, coronary heart disease, blood transfusions, or drugs and food allergies. Physical examination: The abdomen shape was normal and there was no abdominal tenderness or rebound pain. At about 2 cm from the anus, an abscess of 2 cm in diameter at two to three points position was observed (chest-knee position). The abscess surface was ulcerated with a small amount of white pus discharged. The digital anal examination (chest-knee position) indicated an irregular mass which was 2 × 2 × 2 cm in size, and 1 cm away from the anal margin at 2 to 5 points position. The mass was tough with uneven surface and the tenderness was positive. Compression of the mass could induce purulent and mucus discharge from the anal canal mass and perianal abscess. Based on the symptoms and physical examination results, our medical team suspected the diagnosis of anal fistula, but the anal canal cancer or rectal cancer should be excluded.

**Figure 1:**
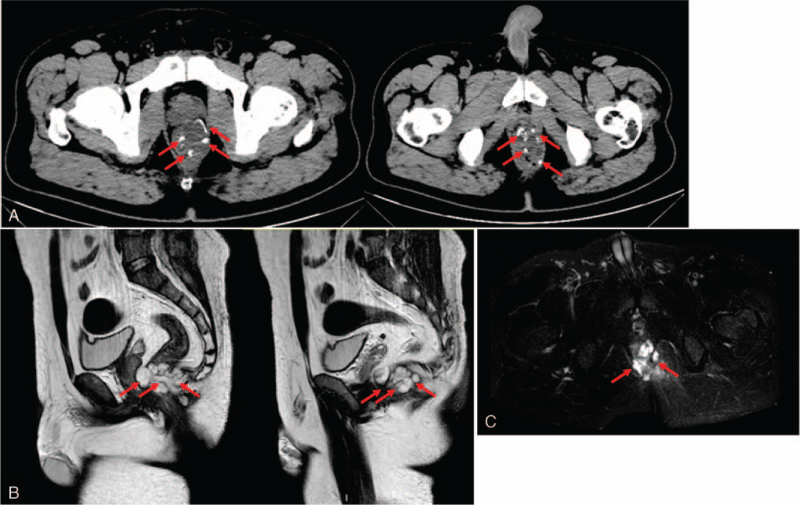
Imageological examination. (A) CT examination indicated the thickened wall of the lower rectum accompanied with a strip-shaped high-density shadow (red arrows). (B) MRI showed a cyst-like mass of about 33 × 57 × 30 mm in the anal area (red arrows). (C) MRI indicated plaque-shaped high signal shadow (red arrows) in the left side of the anus. CT = computer tomography, MRI = magnetic resonance imaging.

The biochemical examination indicated high levels of white blood cells (12.3 × 10^9^/L, normal: 3.5–9.5 × 10^9^/L) and carcinoembryonic antigen (4.62 ng/mL, normal: 0–3 ng/mL). There were no other abnormal biochemical results. The patient didn’t perform the electronic colonoscopy because of the anal stenosis that was caused by the anal fistula surgery. In addition, he also denied the preoperative pathology. The computer tomography (CT) showed that the wall of the lower rectum was thickened and uneven in density. In addition, a strip-shaped high-density shadow can be seen in the rectal wall (Fig. 1A). Chest and abdominal CT examinations didn’t reveal any signs of distant tumor metastasis. The magnetic resonance imaging (MRI) showed a cyst-like mass of about 33 × 57 × 30 mm in the anal area. The lesion penetrated the anal canal, and plaque-shaped high signal shadow can be seen in the left side of the anus (Fig. 1B and C). The imageological examination indicated cystic abnormal signals in the anal area and inflammation responses in the left soft tissue of the anus and the left gluteal muscle.

**Figure 2:**
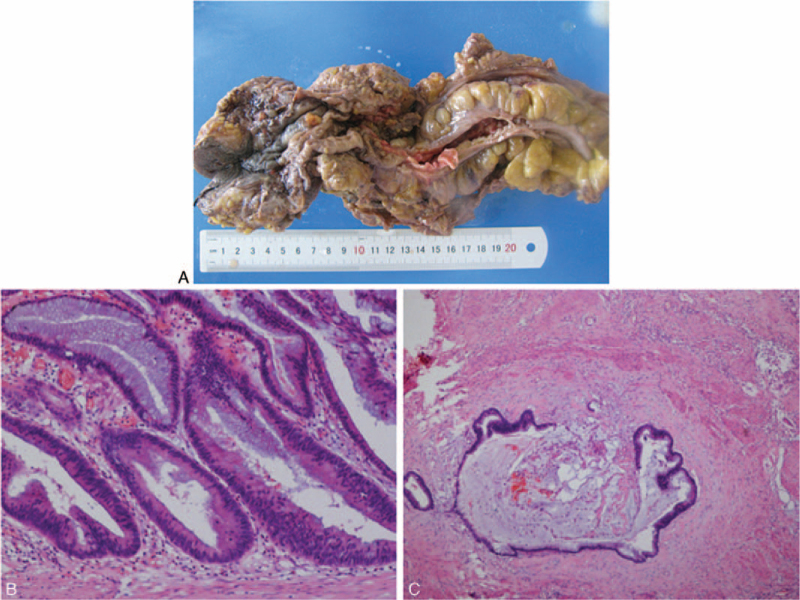
Postsurgical pathology. (A) The resected tumor and nearby tissues. The postsurgical pathology indicated the tumor as anal canal adenocarcinoma (B) (magnification, ×100) with large amounts of mucus (C) (magnification, ×40).

Based on previous examination result, surgical resection was planned for this patient and the specific surgical methods would depend on the intraoperative pathology results. The intraoperative pathology indicated inspected tissue as anal canal adenocarcinoma with heteroepithelium. As a result, the abdominal perineal resection (APR) was performed for this patient. Partial sigmoid colon, total rectum, rectal mesentery, part of the levator ani muscle, anal canal, and perianal canal fat and skin were resected (Fig. 2A). The postsurgical pathology showed that the tumor was anal canal adenocarcinoma with large amounts of mucus (Fig. 2B and C) and was graded as T3N1M0. The patient recovered well and discharged from our department at 12th day postsurgery. This patient received continuous pelvic radiotherapy with 45Gy/25f/5w but refused chemotherapy. The follow-up time was 1 year and no signs of tumor recurrence were found.

## 3 Discussion

Anal canal adenocarcinoma is a kind of malignant tumor that is extremely rare clinically.^[4]^ Most of the tumors are located behind the anal canal and about one-third of the tumors are located outside the anal canal or in the sciatic rectum fossa. The prognostic factors of anal canal adenocarcinoma mainly include the tumor size, tumor staging, surgical methods, whether receiving radiotherapy or chemotherapy, etc.^[1–3]^ About one-half of the tumors are accompanied by anal fistula.^[5,6]^ In this study, the anal canal tumor tissues invaded the whole anal canal and did not locate in the sciatic rectum fossa. This patient mainly complained about the symptoms of anal fistula, which was anal tenderness accompanied by intermittent drainage of mucus discharge. This may because that the tumor tissues invade the anal canal and induce perianal inflammatory responses, thus leading to the formation of anal fistula.

Anal canal adenocarcinoma mainly originates from the anal canal epithelium. The histology and immunophenotype of anal canal adenocarcinoma are the same as those of common colorectal adenocarcinoma, but its location is lower and the prognosis is worse than that of squamous cell carcinoma.^[7,8]^ CT and MRI are important for the diagnosis of anal canal cancer. The electronic colonoscopy and histopathology should be performed for correct diagnosis of anal canal adenocarcinoma. However, because of the anal stenosis that was caused by the anal fistula surgery, the colonoscopy was not performed. In addition, the patient denied the presurgical histopathology examination. The intraoperative histopathology examination was performed which decided further APR surgery for this patient.

Radical resection is important for the treatment of anal canal adenocarcinoma.^[9]^ For early invasive anal canal adenocarcinoma, local resection should be considered. Local resection is less invasive and has a lower incidence of complications. In the opinions of some researchers, local resection is suitable for the treatment of anal canal tumors, which are small in diameter (<2 cm), well-differentiated, and below the dentate line.^[5,7,8]^ In addition, the sphincter should not be invaded. However, if the margin is positive after local resection, radiotherapy or chemotherapy is recommended postsurgery. For patients who are not suitable for local resection (e.g., the sphincter is highly invaded), APR is suggested. For APR, partial sigmoid colon, total rectum, rectal mesentery, part of the levator ani muscle, anal canal, and perianal canal fat and skin should be dissected. Because the rectal vaginal septum is often invaded by tumor tissues, the posterior vaginal wall should also be resected for female patients. The survival time of anal canal cancer is negatively correlated with tumor size. After radical resection, the 5-year survival rate of anal canal cancer is 55% to 71%.^[10]^ The recurrence rate of anal canal cancer after APR is about 40% and the average recurrence time is 12 to 15 months.^[10]^ Local recurrences are the most common and distant metastasis is rare. In our study, the tumor diameter was >2 cm, the tumor penetrated the anal canal, and the sphincter was invaded. As a result, APR was performed.

In recent years, the combination of radiotherapy and chemotherapy attracts more and more attention for the treatment of anal canal cancer.^[11,12]^ In the United States, the comprehensive treatment scheme for anal canal cancer is continuous pelvic radiotherapy, with the total dose of 45 GY (30 GY is total pelvic radiation, 15 GY is true pelvic radiation) and 2 cycles (the 1st and 5th week) of 5-Fu infusion (1000 mg/m^2^), and 1 time of mitomycin (10 mg/m^2^) treatment.^[13–15]^ If the complete remission was not achieved after 6 weeks of treatment, the patient received a 1-week supplementary treatment with 9 GY radiation of primary tumor. If residual lesions are still present, APR is recommended. The 5-year survival rate of patients with anal canal cancer receiving concomitant radiotherapy and chemotherapy is about 73%.^[14]^ Whether radiotherapy and chemotherapy should be performed simultaneously or in sequence is still inconclusive. In addition, whether the combination of radiotherapy and chemotherapy can substitute radical resection is still controversial. In the present study, APR was performed first, followed by radiotherapy.

In this study, we reported a rare case of anal canal adenocarcinoma that was mainly manifested as anal fistula. Comprehensive treatment, including APR and radiotherapy, were performed for this patient. In addition, the characteristics, diagnosis, and treatment methods of anal canal adenocarcinoma were summarized.

## Author contributions

**Conceptualization:** Xiaowei Song, Huimin Zhao, Linxian Zhao.

**Data curation:** Yongping Yang, Linxian Zhao.

**Formal analysis:** Huimin Zhao.

**Funding acquisition:** Jiannan Li.

**Investigation:** Yongqing Zhao.

**Methodology:** Xiaowei Song, Yongping Yang, Yongqing Zhao.

**Software:** Yongping Yang, Linxian Zhao.

**Supervision:** Yongping Yang, Linxian Zhao, Yongqing Zhao.

**Validation:** Linxian Zhao.

**Visualization:** Yongping Yang.

**Writing – original draft:** Xiaowei Song, Jiannan Li.

**Writing – review & editing:** Huimin Zhao, Jiannan Li.
